# Indirect signs of infravesical obstruction on voiding cystourethrography improve post-neonatal posterior urethral valves detection rate

**DOI:** 10.1007/s00330-023-10126-z

**Published:** 2023-08-19

**Authors:** Pierluigi Marzuillo, Maria Paola Belfiore, Anna Di Sessa, Giovanni Torino, Agnese Roberti, Marialuisa Balzano, Alfonso Reginelli, Salvatore Cappabianca, Emanuele Miraglia del Giudice, Angela La Manna, Stefano Guarino, Giovanni Di Iorio

**Affiliations:** 1 Department of Woman, Child and of General and Specialized Surgery, Università Degli Studi Della Campania “Luigi Vanvitelli”, Via Luigi De Crecchio 2, 80138 Naples, Italy; 2 Department of Precision Medicine, Università Degli Studi Della Campania “Luigi Vanvitelli”, Naples, Italy; 3 Pediatric Urology Unit, “Santobono-Pausilipon” Children’s Hospital, Naples, Italy

**Keywords:** Posterior urethral strictures, Diagnostic imaging, Radiography, Contrast media, Cystoscopy

## Abstract

**Objectives:**

To identify the diagnostic performance of clinical and radiological signs (on voiding cystourethrography [VCUG]) to detect posterior urethral valves (PUV) in the post-neonatal period.

**Materials and methods:**

One hundred eighteen males (median age = 0.8 years, range = 1 month–14 years, 48 toilet-trained) undergoing VCUG in a 2-year period were prospectively enrolled. Direct (dilated posterior urethra) and indirect (hypertrophied bladder neck, musculus interuretericus hypertrophy, and trabeculated appearance of the bladder wall) PUV signs on VCUG were assessed. Uroflowmetry was defined pathological by patterns suggesting infravesical obstruction.

**Results:**

Twenty-two patients with direct, 28 with indirect PUV signs on VCUG, and one with normal VCUG but persisting micturition symptoms with pathological uroflowmetry underwent urethrocystoscopy and in 43/51 a PUV diagnosis was made (*n* = 22, 51.2%, with direct PUV signs). In 8/28 patients with indirect signs, PUV were not confirmed. Among non-toilet-trained patients, none of the clinical signs/symptoms was associated with PUV while among toilet-trained patients only pathological uroflowmetry (odds ratio, OR = 4.0 [95% confidence interval:1.2–13.2; *p* = 0.02]) and pathological uroflowmetry with history of urinary tract infection (OR = infinity) were significantly associated with PUV. Significant associations with PUV of direct and indirect signs on VCUG were found both in toilet-trained and non-toilet trained patients. Direct PUV sign had 100% specificity and sensitivity while indirect PUV signs showed sensitivity = 58.1% and specificity = 89.3%. The absence of any radiological sign had a negative predictive value = 98.5%.

**Conclusion:**

Only half of patients with endoscopy-confirmed PUV presents with direct sign of PUV on VCUG. Accounting for indirect PUV signs on VCUG and pathological uroflowmetry (in toilet-trained children) could improve the PUV detection rate.

**Clinical relevance statement:**

Indirect radiological PUV signs should be valorized when interpreting VCUG to improve the PUV detection rate. The absence of any radiological PUV (direct and indirect) sign on VCUG excludes PUV with a very high negative predictive value.

**Key Points:**

*• Worldwide agreement is that a non-dilated urethra on voiding cystourethrography excludes obstruction.*

*• Half of patients with posterior urethral valves have non-dilated urethra on voiding cystourethrography.*

*• Accounting for indirect signs of posterior urethral valves on voiding cystourethrography improves the diagnostic performance.*

**Supplementary Information:**

The online version contains supplementary material available at 10.1007/s00330-023-10126-z.

## Introduction

Posterior urethral valves (PUV) are the main cause of lower urinary tract obstruction in boys [1]. They are responsible for 17% of cases of end-stage renal disease in childhood and might also cause voiding symptoms in older children [2].

Most cases are identified in the prenatal or neonatal period [1, 3]; however, up to 34% of patients receive a late diagnosis [3].

The variable rates of PUV detection could be the consequence of lack of clinical and/or radiological reference standards [4]. Urethrocystoscopy is the gold-standard for the PUV diagnosis [5], but, before referring a child to urethrocystoscopy, a voiding cystourethrography (VCUG) is routinely made [5].

Worldwide agreement is that if an urethra appears as non-dilated on VCUG, an obstruction can be excluded [4]. Recent reports, however, indicate that preoperative VCUG suspicion of PUV was present in only 46% of non-toilet trained [2] and 59% of toilet-trained patients with PUV [6].

On the other hand, it has been shown that the presence of indirect radiological findings of infravesical obstruction is common in patients with endoscopy-confirmed PUV and urethra appearing non-dilated on VCUG [2].

The idea of this study was derived from the online-first publication of the study by Haid et al. in March 2020 [2], and this idea was based on the hypothesis that indirect radiological signs of infravesical obstruction on VCUG could facilitate the diagnosis of PUV and could increase the detection rate of PUV without classical prenatal/neonatal presentation.

Therefore, we aimed at identifying the diagnostic performance of clinical and radiological VCUG signs for urethrocystoscopy-confirmed PUV diagnosis in a population of boys aged 1 month–14 years.

## Methods

We prospectively enrolled all the boys consecutively undergoing VCUG for any reason between May 2020 and May 2022. Our institutional review board approved the study (file number 371). The patients were recruited in a center, lacking of neonatal unit, for the management of congenital anomalies of the kidney and urinary tract (CAKUT) and lower urinary tract symptoms in children.

Inclusion criteria: (i) male gender; (ii) 1 month–14 years of age; (iii) undergoing VCUG for any reason.

Exclusion criteria: (i) denied consent to participate; (ii) previously detected congenital anomalies of the urethra.

### Protocol of the study

VCUG was performed in case of (1) recurrent (febrile or not) urinary tract infections (UTI); (2) first febrile UTI by a non-*Escherichia coli* bacterium; (3) mono- or bilateral megaureter > 7 mm of diameter; (4) mono- or bilateral hydronephrosis with antero-posterior diameter of the pelvis (APDP) > 15 mm; (5) small kidney (kidney length < 2 standard deviation score) and/or mono/bilateral renal dysplasia (cortical thinning, poor corticomedullary differentiation, renal cysts) detected with ultrasound; (6) vesico-ureteral reflux (VUR) needing of follow-up VCUG; and (7) micturition symptoms and uroflowmetry suggestive of infravesical obstruction.

M.P.B. performed the VCUGs according to a standard protocol [7]. All the images were collegially reviewed to select patients needing urethrocystoscopy. G.D.I. performed the urethrocystoscopies.

Patients with small kidneys also underwent Tc99mMag3 scintigraphy (Mag3S) to evaluate the split renal function of the kidneys.

### Definition of PUV signs on VCUG

**Fig. 1:**
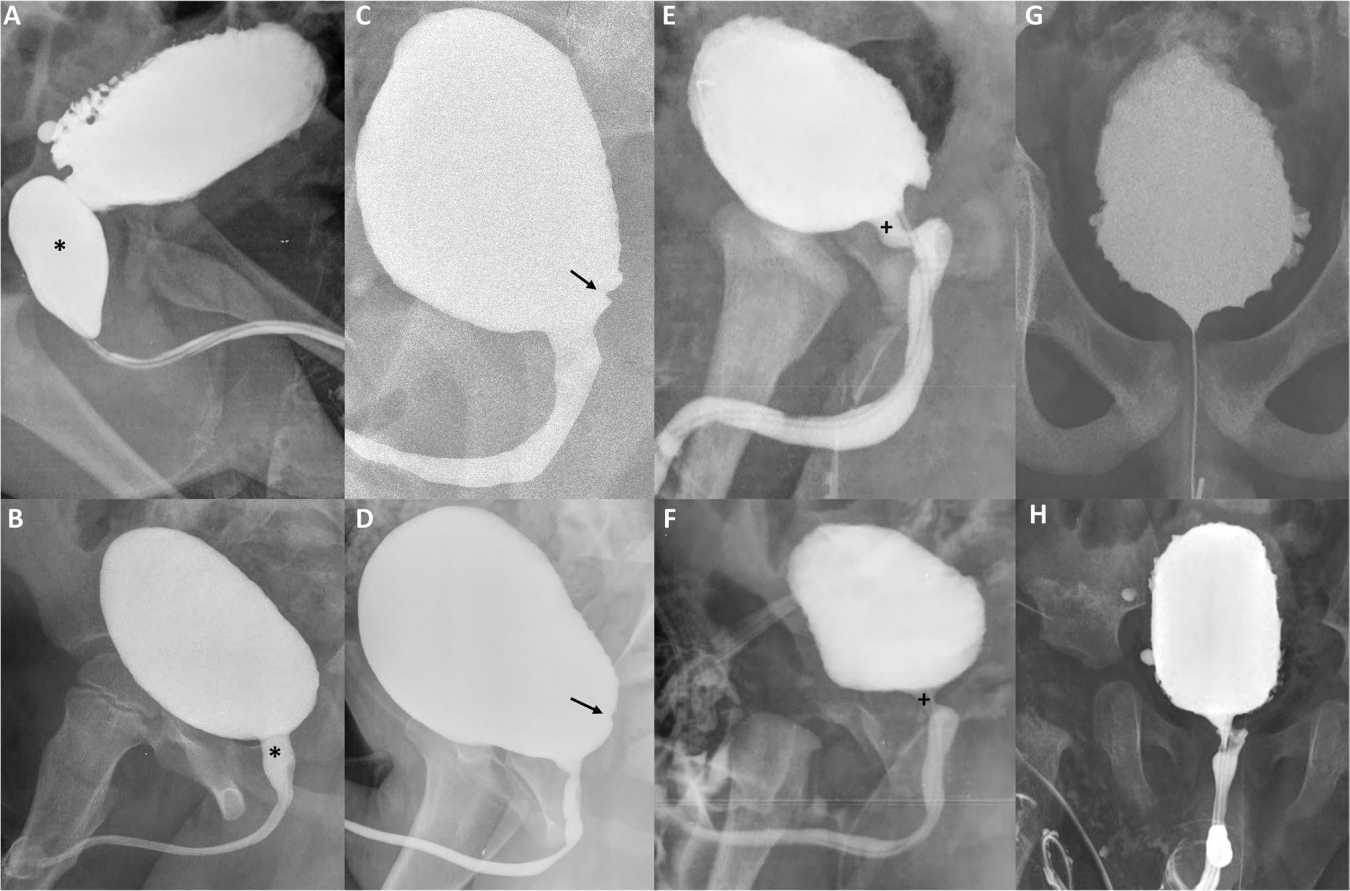
Direct and indirect PUV signs. **A** and **B** show direct PUV signs. **C**, **D**, **E**, **F**, **G**, and **H** show indirect PUV signs. In **A** and **B**, a dilated posterior urethra is evident (asterisks). In **C** and **D**, the musculus interuretericus hypertrophy is indicated by the arrow. In **E** and **F**, the plus symbol underlines the hypertrophied bladder neck. In **G** and **H**, trabeculated appearance of the bladder wall is shown

Direct PUV sign: dilated posterior urethra [2, 6] (Fig. 1A and B).

Indirect PUV signs: musculus interuretericus hypertrophy (indentation at the level of the ureteral orifices on lateral/oblique pictures either during bladder filling or voiding) (Fig. 1C and D), hypertrophied bladder neck (unusual indentation of the bladder neck on lateral/oblique pictures during voiding) (Fig. 1E and F), and trabeculated appearance of the bladder wall (toothed aspect of the bladder wall concerning the pars fixa dorsally during filling or voiding or the pars libera during filling as a sign of hypertrophy of the detrusor muscle) (Fig. 1G and H) [2]. We considered the presence of bladder wall diverticula as part of trabeculations.

### Other definitions

**Fig. 2:**
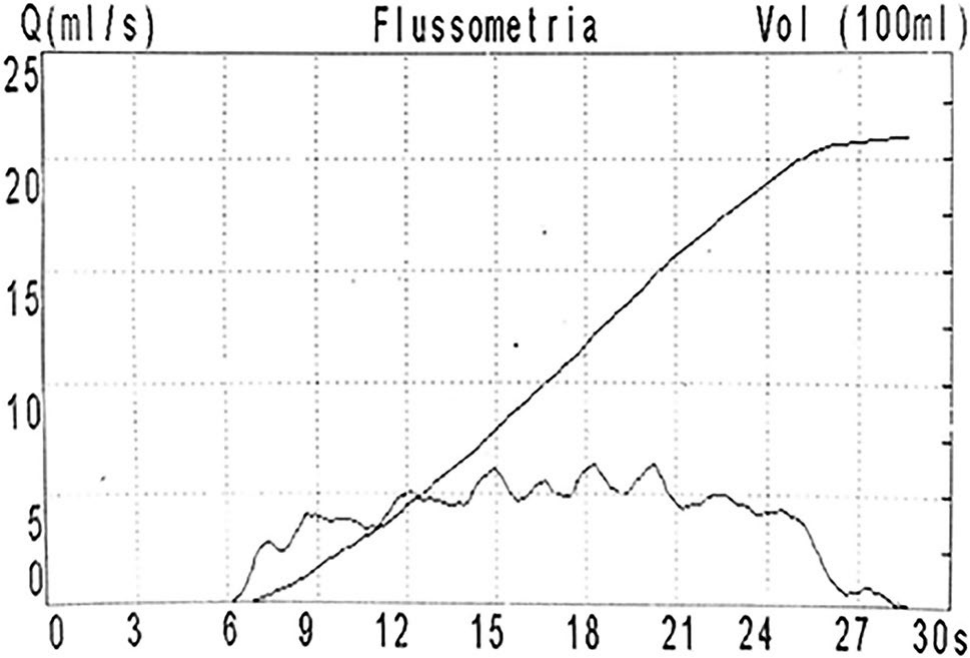
Uroflowmetry suggesting infravesical obstruction. This is a plateau-shaped uroflow curve pattern. It indicates an infravesical obstruction and is characterized by a flattened and low-amplitude prolonged flow curve

Pathological uroflowmetry: anomalies of at least two uroflowmetries suggesting infravesical obstruction such as plateau-shaped curve pattern (Fig. 2) and/or maximum urinary flow rate (Qmax) < 5th percentile for age, gender, and urine volume [8, 9].

Micturition symptoms: day-time incontinence and/or straining to void and/or hesitancy and/or urgency and/or frequency with/without nocturnal enuresis [9].

Kidney injury: presence of either hypertension, or proteinuria, or reduced estimated glomerular filtration rate [10] according to the specific reference values for age [11].

### Statistical analysis

*p* values < 0.05 were considered significant. Differences for continuous variables were analyzed with the independent-sample *t* test in case of normality and with the Mann–Whitney test in case of non-normality. Non-continuous variables were compared by the chi-squared test or Fisher’s exact test.

Logistic regression was used to calculate the odds ratio (OR) of presenting PUV. Clinical and radiological predictors were separately analyzed. In univariate analysis, the Bonferroni correction was applied, and only if 2 or more variables were significant after Bonferroni correction, the multivariate analysis was run. The variables with OR = infinity were not added into the multivariate analysis.

We calculated sensitivity, specificity, accuracy, positive and negative predictive value (PPV and NPV), and positive and negative likelihood ratio (PLR and NLR) toward urethrocystoscopy confirmed PUV of the direct and indirect PUV signs on VCUG.

The SPSS and MedCalc software for Windows were used for all statistical analyses.

## Results

### General characteristics

We enrolled 118 males undergoing VCUG. Forty-eight of them (40.7%) were toilet-trained. The median age on VCUG was 0.8 years (interquartile range, IQR, 5.6). In particular, the age was 0.4 years (IQR = 0.5) among non-toilet-trained and 7.0 years (IQR = 5.8) among toilet-trained patients.

Forty-three patients (36.4%) presented a final PUV diagnosis after urethrocystoscopy. Respectively 25 (58.1%) and 18 (41.9%) out of 43 patients with PUV were toilet- and non-toilet-trained.

The general characteristics of the enrolled population are shown in Table 1.

**Table 1:** Clinical, radiological, and biochemical characteristics of all enrolled patients, and of the patients with and without PUV

	All patients No. = 118	PUV (no) No. = 75	PUV (yes) No. = 43	*p*
Age at cystography, yr, median (IQR)	0.8 (5.6)	0.6 (2.3)	4.7 (8.5)	< 0.001
Birth weight, kg, mean (SDS)	3.2 (0.5)	3.2 (0.5)	3.2 (0.5)	0.54
Birth weight < 2500 gr, no. (%)	13 (11.0)	7 (9.3)	6 (13.9)	0.59
Preterm birth, no. (%)	20 (16.9)	12 (16.0)	8 (18.6)	0.13
Being toilet-trained, no. (%)	48 (40.7)	23 (30.7)	25 (58.1)	0.003
Micturition symptoms, no. (%)	24 (20.3)	8 (10.6)	16 (37.2)	< 0.001
Febrile UTI, no. (%)	43 (36.4)	26 (34.7)	17 (39.5)	0.59
Non-febrile UTI, no. (%)	22 (18.6)	11 (14.7)	10 (23.2)	0.24
UTI and UTD, no. (%)	21 (17.8)	14 (18.7)	7 (16.3)	0.81
UTI without VUR, no. (%)	26 (22.0)	14 (18.7)	12 (27.9)	0.24
UTI with VUR, no. (%)	27 (22.9)	17 (22.7)	10 (23.3)	0.005
Pathological uroflowmetry, no. (%)	25 (21.2)	8 (10.7)	17 (39.5)	< 0.001
Pathological uroflowmetry with history of UTI, no. (%)	8 (6.8)	0 (0)	8 (18.6)	< 0.001
Monolateral APDP > 15 mm, no. (%)	18 (15.2)	14 (18.7)	4 (9.3)	0.17
Bilateral APDP > 5 mm, no. (%)	29 (24.6)	20 (26.7)	9 (20.9)	0.48
Megaureter > 7 mm, no. (%)	22 (18.6)	14 (18.7)	8 (18.6)	0.99
Bilateral megaureter, no. (%)	5 (4.2)	2 (2.7)	3 (6.9)	0.35
PUV direct signs on VCUG, no. (%)	22 (18.6)	0 (0)	22 (51.2)	< 0.001
PUV indirect signs on VCUG, no. (%)	33 (27.9)	8 (10.6)	25 (58.1)	< 0.001
PUV direct and indirect signs, no. (%)	5 (4.2)	0 (0)	5 (11.6)	0.005
Any radiological sign, no. (%)	50 (42.4)	8 (10.6)	42 (97.7)	< 0.001
Presence of VUR, no. (%)	39 (33.0)	26 (34.7)	13 (30.2)	0.62
KL < 2SDS, no. (%)	17 (14.4)	9 (12.0)	8 (18.6)	0.32
SRF < 40%, no. (%)	19 (16.1)	12 (16)	7 (16.3)	0.96
Hypertension, no. (%)	0 (0)	0 (0)	0 (0)	0.99
Reduced eGFR, no. (%)	5 (4.2)	3 (4.0)	2 (4.6)	0.99
Proteinuria, no. (%)	3 (2.5)	2 (2.7)	1 (2.3)	0.99
Kidney injury, no. (%)	8 (6.8)	5 (6.7)	3 (7.0)	0.99

For normal distributed variables, means ± SDS are shown, while for non-parametric ones, median and interquartile range are shown

*Abbreviations: APDP*, antero-posterior diameter of the pelvis; *eGFR*, estimated glomerular filtration rate; *IQR*, interquartile range; *KL*, kidney length; *PUV*, posterior urethral valves; *SDS*, standard deviation score; *SRF*, split renal function; *UTD*, urinary tract dilation; *UTI*, urinary tract infection; *VCUG*, voiding cystourethrography; *VUR*, vesico-ureteral reflux

### Indications to VCUG

Most of the 48 toilet-trained patients underwent VCUG for micturition symptoms and pathological uroflowmetry, for UTI, or for already known VUR needing of follow-up VCUG, while most of the 70 non-toilet-trained patients underwent VCUG for pelvic or ureteric dilation and febrile UTI (Table 2).

**Table 2:** Indications to VCUG execution

Toilet-trained patients (*n* = 48)		Non-toilet-trained patients (*n* = 70)	
Indication	*n* (%)	Indication	*n* (%)
Micturition symptoms and pathological uroflowmetry	22 (45.8)	Febrile UTI determined by a non-coli bacterium	22 (31.4)
Already known VUR needing of follow-up VCUG	10 (20.8)	Hydronephrosis	20 (28.6)
Febrile UTI determined by a non-coli bacterium	6 (12.5)	Megaureter	13 (18.6)
Recurrent non-febrile UTIs	5 (10.4)	Recurrent febrile UTIs	9 (12.8)
Recurrent febrile UTIs	2 (4.2)	Recurrent non-febrile UTIs	4 (5.7)
Micturition symptoms associated with recurrent febrile/not febrile UTIs	2 (4.2)	Small kidney	2 (2.9)
Megaureter	1 (2.1)		

*Abbreviations: UTI*, urinary tract infection; *VCUG*, voiding cystourethrography; *VUR*, vesico-ureteral reflux

### VCUG findings of PUV patients

Seventeen patients showed only direct signs of PUV, 28 only indirect signs, and 5 with both. The VCUG-found indirect VUP signs are shown in Table 3.

**Table 3:** Indirect PUV signs on VCUG

Findings	Only indirect PUV signs (*n* = 28)	Direct and indirect PUV signs (*n* = 5)
Hypertrophied bladder neck, *n* (%)	8 (28.6)	0 (0)
Musculus interuretericus hypertrophy, *n* (%)	8 (28.6)	0 (0)
Trabeculated appearance of the bladder wall, *n* (%)	7 (25.0)	3 (60)
Trabeculated appearance of the bladder wall and hypertrophied bladder neck, *n* (%)	3 (10.7)	2 (40)
Trabeculated appearance of the bladder wall and hypertrophied musculus interuretericus, *n* (%)	2 (7.1)	0 (0)

*Abbreviation*: *VCUG*, voiding cystourethrography; *PUV*, posterior urethral valves

### Urethrocystoscopy detection of PUV

All 50 patients with direct and/or indirect PUV signs underwent urethrocystoscopy. Also, one patient without any PUV sign on VCUG but with persisting day-time incontinence, persisting reduced Qmax, and prolonged flow underwent this procedure.

A PUV diagnosis was made in 43 out of 51 (84.3%) patients. No other urethral abnormalities were found. Among these 43 patients, direct PUV signs were evident in 22 (51.2%). In all the patients with direct and in 20 out of 28 patients (71.4%) with indirect PUV signs on VCUG, PUV were found on urethrocystoscopy. PUV was also detected in the only patient with normal VCUG.

The 8 patients without urethrocystoscopic PUV confirmation (all of them toilet-trained) presented hypertrophied bladder neck (*n* = 4), musculus interuretericus hypertrophy (*n* = 3), and trabeculated appearance of the bladder wall (*n* = 1) on VCUG. All these patients presented initially persistently reduced Qmax with flow normalization after 3–6 months of urotherapy.

### Clinical predictors of PUV

Among non-toilet-trained patients, none of the examined clinical factors was significantly associated with PUV (Supplementary Table 1).

Among toilet-trained patients, micturition symptoms and pathological uroflowmetry were associated with PUV. The association of pathological uroflowmetry increased in case of concomitant history of febrile or not-febrile UTI (Supplementary Table 2).

In toilet-trained patients, the exploratory univariate analysis evaluating the OR of presenting PUV showed that a pathological uroflowmetry with history of UTI had OR = infinity, the presence of micturition symptoms had OR = 3.3 (95% confidence interval (CI): 1.02/11.0; *p* = 0.046), and a pathological uroflowmetry showed an OR = 4.0 (95%CI: 1.2/13.2; *p* = 0.02). The multivariate analysis was not run because only pathological uroflowmetry had significant *p* after Bonferroni correction (*p* < 0.025).

### VCUG predictors of PUV

A significant association with PUV of direct and/or indirect PUV signs on VCUG was found both among non-toilet- (Supplementary Table 1) and toilet-trained patients (Supplementary Table 2).

Direct PUV signs showed OR = infinity in both groups, while indirect PUV signs had an OR of showing urethrocystoscopy-confirmed PUV of 18.8 (95%CI: 4.9–72.2; *p* < 0.001) among non-toilet trained patients and of 7.2 (95%CI: 1.7–30.6; *p* = 0.007) among toilet-trained patients.

### Diagnostic accuracy of direct and indirect PUV signs on VCUG

The direct PUV sign had 100% specificity and PPV and infinity PLR while the indirect PUV signs showed sensitivity = 58.1%, specificity = 89.3%, and PLR = 5.4 (Table 4). Evaluating any PUV sign, the prognostic accuracy toward PUV reached sensitivity = 97.7%, specificity = 89.3%, and NPV = 98.5% (Table 4).

**Table 4:** Prognostic accuracy of direct and indirect PUV signs on VCUG

	True positive:false positive	True negative:false negative	Sensitivity (95%CI)	Specificity (95%CI)	Accuracy (95%CI)	Positive likelihood ratio (95%CI)	Negative likelihood ratio (95%CI)	Positive predictive value (95%CI)	Negative predictive value (95%CI)
Direct PUV signs	22:0	75:21	51.2% (35.5–66.7%)	100% (92.5–100%)	82.2% (74.1–88.6%)	Infinity*	0.49 (0.36–066)	100%	78.1% (72.4–82.9%)
Indirect PUV signs	25:8	67:18	58.1% (42.1–72.9%)	89.3% (80.1–95.3%)	78.0% (69.4–85.1%)	5.4 (2.7–11.0)	0.47 (0.3–0.7)	75.8% (60.8–86.3%)	78.8 (72.2–84.2)
Any radiological sign	42:8	67:1	97.7% (87.7–99.9%)	89.3 (80.1–95.3%)	92.4% (86.0–96.4%)	9.2 (4.8–17.7)	0.03 (0.0–0.2)	84.0% (73.1–91.0%)	98.5% (90.6–99.8%)

^*^All the patients with direct PUV signs showed PUV confirmation at urethrocystoscopy

*Abbreviations: CI*, confidence interval; *PUV*, posterior urethral valves; *VCUG*, voiding cystourethrography

## Discussion

This is the first prospective study evaluating clinical and radiological predictors of urethrocystoscopy-confirmed PUV in children. Timely diagnoses and especially no missed radiological signs allowing to suspect PUV following VCUG are mandatory to optimize invasive procedures in childhood. The VCUG, in fact, has high biological costs exposing children both to pain due to urethral catheterization and to non-negligible doses of X-rays [7]. Furthermore, no missed PUV diagnoses could be important to prevent future development of chronic kidney disease as consequence of the obstruction [12] and its related social costs [13]. Finally, missed PUV and PUV showing late-presentation may also cause very troubling voiding symptoms in older children [6]. Also when symptoms are subtle, detrusor anomalies could be progressive and may be associated with kidney injury, both persisting also after valve ablation (late onset PUV are not minor) [12].

In this view, also other studies searched for different findings allowing optimization of VCUG interpretation such as bladder and posterior urethra height to width ratio [14, 15].

Recent data showed unsuspicious urethra on VGUG respectively in 54.3% of children with a mean age of 27 months [2], and in 41.2% of boys with symptomatic late-presenting (at mean age of 7.3 years) PUV [6]. These studies enrolled children with an already known PUV diagnosis on urethrocystoscopy and retrospectively evaluated the clinical and radiological data [2, 6]. We enrolled all the patients undergoing VCUG in a 2-year period to explore associations of clinical and radiological PUV signs with urethrocystoscopy-confirmed PUV diagnosis to mimic the daily challenges during the decision-process leading to urethrocystoscopy execution.

While among non-toilet-trained patients (after the neonatal period) none of the clinical parameters was predictive of PUV, among toilet-trained patients only pathological uroflowmetry was predictive. The association of pathological uroflowmetry with PUV increased in case of concomitant history of UTI. On this respect, it has to be underlined that recurrent non-febrile UTIs in boys have a different and higher relevance than in girls.

The dilation of the posterior urethra (direct PUV sign) on VCUG was highly predictive of PUV with specificity and PPV of 100%. In line with the previous literature [2, 6], only 51.2% of the patients with endoscopically detected PUV presented PUV direct signs on VCUG. This further emphasizes the need to improve the VCUG interpretation and the selection of patients to refer to urethrocystoscopy. In this view, the indirect PUV signs appear to be promising. In fact, we referred all the patients with indirect PUV signs to urethrocystoscopy and we observed that PUV were confirmed in 20 out of 28 patients (71.4%) with only indirect signs. The absence of any PUV sign on VCUG showed a very high NPV (98.5%).

On the other hand, urethrocystoscopy was negative in 8 patients referred based on VCUG results. They showed initially persistently very low Qmax with indirect PUV signs on VCUG and during follow-up they experienced uroflowmetry normalization as consequence of urotherapy. To optimize the indications to VCUG, we believe that before performing VCUG in patients with pathological uroflowmetry, the exam after urotherapy should be repeated.

The only patient with persisting micturition symptoms and persisting very low Qmax without direct/indirect PUV signs on VCUG underwent urethrocystoscopy. After 6 months of unsuccessful treatment with oxybutynin, the persistence of low Qmax and of micturition symptoms was considered sufficient to perform urethrocystoscopy despite normal VCUG. Interestingly, PUV were found at the endoscopy. This is in line with some of the cases described by Ozen et al [6], and it supports our hypothesis that urethrocystoscopy without performing VCUG could be immediately performed when there is a strong clinical indication for the presence of valves.

Moreover, it is important to note that our practice lacks of neonatal unit and we usually enroll and manage only children with CAKUT aged ≥ 1 month and this explains the lack of enrollment of patients with the classical prenatal/neonatal PUV presentation and why some of the signs classically related to VUP (i.e., megaureter) did not result associated with VUP in our cohort. On the other hand, our work setting allows to focus on a population of patients without the classical PUV presentation providing new information about VCUG interpretation outside the neonatal period.

Voiding symptoms had prevalence of 5.4% among the 92 patients of the Haid et al. [2] series compared to prevalence of 50% among the toilet-trained patients of our series. Moreover, we also found higher median age in patients with PUV compared with those without PUV. This is probably because our setting determined the enrollment of a significant number of patients aged > 2 years and of toilet-trained patients.

Our data, however, suggest that there is a high percentage of voiding symptoms in toilet-trained patients with delayed presentation of PUV.

Noteworthy, we enrolled 10 patients with an already known VUR needing a follow-up VCUG. Interestingly, in 4 of these patients, we were able to detect indirect signs of PUV only at the follow-up VCUG when our awareness about the importance of PUV indirect signs on VCUG had increased [2, 6]. This could also underline that a specific training to correctly identify indirect signs (especially the musculus interuretericus hypertrophy) is required because these signs are not always easily identifiable.

The main limitation of this study is that not all the enrolled patients underwent an urethrocystoscopy independently from clinical and radiological findings. However, this approach — without a clinical correlate — would be unethical because the urethrocystoscopy in children is an invasive procedure requiring sedation.

In conclusion, giving value to indirect PUV signs on VCUG, the predictivity of VCUG toward urethrocystoscopy-confirmed PUV increases and the percentage of missed PUV reduces in children with age ≥ 1 month. In addition, the absence of any (direct and/or indirect) PUV sign on VCUG provides a very high NPV. In toilet-trained patients, the presence of pathological uroflowmetry, especially if associated with history of UTI, could help in the selection of patients to refer to VCUG and — in selected cases — to urethrocystoscopy if VCUG is normal.

### Supplementary Information

Below is the link to the electronic supplementary material.

Supplementary file1 (PDF 31 KB)

## Abbreviations

APDP: Antero-posterior diameter of the pelvis
CAKUT: Congenital anomalies of the kidney and urinary tract
IQR: Interquartile range
Mag3S: Tc99mMag3 scintigraphy
NLR: Negative likelihood ratio
NPV: Negative predictive value
OR: Odds ratio
PLR: Positive likelihood ratio
PPV: Positive predictive value
PUV: Posterior urethral valves
Qmax: Maximum urinary flow rate
UTI: Urinary tract infection
VCUG: Voiding cystourethrography
VUR: Vesico-ureteral reflux

## Declarations

### Guarantor

The scientific guarantor of this publication is Pierluigi Marzuillo.

### Statistics and biometry

One of the authors has significant statistical expertise.

### Methodology

• Prospective

• diagnostic or prognostic observational study

• multicenter study
