# Supplementary material for: Indirect signs of infravesical obstruction on voiding cystourethrography improve post-neonatal posterior urethral valves detection rate
Source: Eur Radiol. 2023 Aug 19;34(2):780–7. doi: 10.1007/s00330-023-10126-z (PMC10853084; doi:10.1007/s00330-023-10126-z)
Supplement: Supplementary file 1 — Supplementary file1 (PDF 31 KB) [file 330_2023_10126_MOESM1_ESM.pdf]

Indirect signs of infravesical obstruction on voiding cystourethrography improve post-neonatal posterior urethral valves detection rate

Electronic Supplementary Material

Supplementary Table 1. Clinical, radiological, and biochemical characteristics of the non-toilet-trained patients, with and without PUV.

	All patients No.= 70	PUV (no) No.= 52	PUV (yes) No.= 18	p
**Age at cystography, yr, median (IQR)**	0.40 (0.50)	0.39 (0.47)	0.42 (0.66)	0.65
**Birth weight, kg, mean (SDS)**	3.2 (0.58)	3.2 (0.57)	3.2 (0.63)	0.88
**Birth weight <2500gr, No. (%)**	7 (10.0)	4 (7.7)	3 (16.7)	0.32
**Preterm birth, No. (%)**	13 (18.6)	9 (17.3)	4 (22.2)	0.73
**Febrile UTI, No. (%)**	30 (42.8)	21 (40.4)	9 (50.0)	0.5
**Non-febrile UTI, No. (%)**	9 (12.8)	7 (13.5)	2 (11.1)	0.99
**UTI and UTD, No. (%)**	17 (24.3)	11 (21.1)	6 (33.3)	0.29
**UTI without VUR, No. (%)**	18 (25.7)	12 (23.1)	6 (33.3)	0.22
**UTI with VUR, No. (%)**	16 (22.8)	12 (23.1)	4 (22.2)	0.99
**Monolateral APDP>15mm, No. (%)**	13 (18.6)	11 (21.1)	2 (11.1)	0.49
**Bilateral APDP>5mm, No. (%)**	23 (32.8)	17 (32.7)	6 (33.3)	0.96
**Megaureter>7mm, No. (%)**	18 (25.7)	12 (23.1)	6 (33.3)	0.39
**Bilateral megaureter, No. (%)**	3 (4.3)	1 (1.9)	2 (11.1)	0.09
**PUV direct signs on VCUG, No. (%)**	7 (10)	0 (0)	7 (38.9)	<0.001
**PUV indirect signs on VCUG, No. (%)**	17 (24.2)	5 (9.6)	12 (66.7)	<0.001
**PUV direct and indirect signs, No. (%)**	1 (1.4)	0 (0)	1 (5.6)	0.25
**Any radiological sign, No. (%)**	23 (32.8)	5 (9.6)	18 (100.0)	<0.001
**Presence of VUR, No. (%)**	25 (35.7)	19 (36.5)	6 (33.3)	0.80
**KL<2SDS, No. (%)**	8 (11.4)	5 (9.6)	3 (16.6)	0.41
**SRF<40%, No. (%)**	13 (18.6)	10 (19.2)	3 (16.7)	0.81
**Hypertension, No. (%)**	0 (0)	0 (0)	0 (0)	0.99
**Reduced eGFR, No. (%)**	0 (0)	0 (0)	0 (0)	0.99
**Proteinuria, No. (%)**	2 (2.9)	2 (3.8)	0 (0)	0.99
**Kidney injury, No. (%)**	2 (2.9)	2 (3.8)	0 (0)	0.99

For normal distributed variables means ± SDS are shown, while for non-parametric ones median and interquartile range are shown.

Abbreviations: APDP, antero-posterior diameter of the pelvis; eGFR, estimated glomerular filtration rate; IQR, interquartile range; KL, kidney length; PUV, posterior urethral valves; SDS, standard deviation score; SRF, split renal function; UTD, urinary tract dilation; UTI, urinary tract infection; VCUG, voiding cystourethrography; VUR, vesico-ureteral reflux.

Supplementary Table 2. Clinical, radiological, and biochemical characteristics of the toilet-trained patients with and without PUV.

	All patients No.= 48	PUV (no) No.= 23	PUV (yes) No.= 25	p
**Age at cystography, yr, mean (SDS)**	7.4 (4.1)	6.5 (4.3)	8.3 (3.8)	0.13
**Birth weight, kg, mean, (SDS)**	3.1 (0.47)	3.0 (0.46)	3.1 (0.49)	0.57
**Birth weight <2500gr, No. (%)**	6 (12.5)	3 (13.0)	3 (12.0)	0.99
**Preterm birth, No. (%)**	7 (14.6)	3 (13.0)	4 (16.0)	0.99
**Micturition symptoms, No. (%)**	24 (50.0)	8 (34.8)	16 (64.0)	0.04
**Febrile UTI, No. (%)**	13 (27.1)	5 (21.7)	8 (32.0)	0.42
**Non-febrile UTI, No. (%)**	12 (25.0)	4 (17.4)	8 (32.0)	0.32
**UTI and UTD, No. (%)**	4 (8.3)	3 (13.0)	1 (4.0)	0.34
**UTI without VUR, No. (%)**	8 (16.7)	2 (8.7)	6 (24.0)	0.25
**UTI with VUR, No. (%)**	11 (22.9)	5 (21.7)	6 (24.0)	0.99
**Pathological uroflowmetry, No. (%)**	25 (52.1)	8 (34.8)	17 (68.0)	0.02
**Pathological uroflowmetry without UTI, No. (%)**	14 (29.2)	4 (17.4)	10 (40.0)	0.12
**Pathological uroflowmetry with UTI, No. (%)**	8 (16.7)	0 (0)	8 (32.0)	0.004
**Monolateral APDP>15mm, No. (%)**	5 (10.4)	3 (13.0)	2 (8.0)	0.66
**Bilateral APDP>5mm, No. (%)**	6 (12.5)	3 (13.0)	3 (12.0)	0.99
**Megaureter>7mm, No. (%)**	4 (8.3)	2 (8.7)	2 (8.0)	0.99
**Bilateral megaureter, No. (%)**	2 (4.2)	1 (4.3)	1 (4.0)	0.99
**PUV direct signs on VCUG, No. (%)**	15 (31.2)	0 (0)	15 (60.0)	<0.001
**PUV indirect signs on VCUG, No. (%)**	16 (33.3)	3 (13.0)	13 (52.0)	0.006
**PUV direct and indirect signs, No. (%)**	4 (8.3)	0 (0)	4 (16.0)	0.11
**Any radiological sign, No. (%)**	27 (56.3)	3 (13.0)	24 (96.0)	<0.001
**Presence of VUR, No. (%)**	14 (29.2)	7 (30.4)	7 (28.0)	0.85
**KL<2SDS, No. (%)**	9 (18.9)	4 (17.4)	5 (20.0)	0.99
**SRF<40%, No. (%)**	6 (12.5)	2 (8.7)	4 (16.0)	0.67
**Hypertension, No. (%)**	0 (0)	0 (0)	0 (0)	0.99
**Reduced eGFR, No. (%)**	5 (10.4)	3 (13.0)	2 (8.0)	0.66
**Proteinuria, No. (%)**	1 (2.1)	0 (0)	1 (4.0)	0.99
**Kidney injury, No. (%)**	6 (12.5)	3 (13.0)	3 (12.0)	0.99

For normal distributed variables means ± SDS are shown, while for non-parametric ones median and interquartile range are shown.

Abbreviations: APDP, antero-posterior diameter of the pelvis; eGFR, estimated glomerular filtration rate; IQR, interquartile range; KL, kidney length; PUV, posterior urethral valves; SDS, standard deviation score; SRF, split renal function; UTD, urinary tract dilation; UTI, urinary tract infection; VCUG, voiding cystourethrography; VUR, vesico-ureteral reflux.

Eur Radiol(2023) Marzuillo P, Belfiore MP, Di Sessa A et al.
